# Simultaneous removal of SO_2_ and NO_*x*_ from flue gas by low-temperature adsorption over activated carbon

**DOI:** 10.1038/s41598-021-90532-9

**Published:** 2021-05-26

**Authors:** Shiqing Wang, Shisen Xu, Shiwang Gao, Ping Xiao, Minhua Jiang, He Zhao, Bin Huang, Lianbo Liu, Hongwei Niu, Jinyi Wang, Dongfang Guo

**Affiliations:** 1 Huaneng Clean Energy Research Institute, Beijing, 102209 China; 2 China Huaneng Group Co., Ltd., Beijing, 100031 China; 3 Beijing Key Laboratory of CO2 Capture and Process, Beijing, 102209 China; 4 State Key Laboratory of Coal Based Clean Energy, Beijing, 102209 China

**Keywords:** Environmental sciences, Energy science and technology

## Abstract

An exceptional phenomenon has been observed that SO_2_ and NO_*x*_ in flue gas can be effectively adsorbed over activated carbon with a surprising capacity at cold temperatures with the presence of oxygen. In this study, the adsorption characteristics of NO and SO_2_ over activated carbon at 80, 20, 0, and − 20 is experimentally investigated. Without the presence of oxygen, adsorption of NO is negligible. In the presence of oxygen, NO can be oxidized to NO_2_ over activated carbon which leads to the co-adsorption of NO/NO_2_ within the adsorption bed. Catalytic oxidation of NO over activated carbon can be significantly enhanced at cold temperatures, leading to an extraordinary increase of adsorption capacity of NO. With an initial concentration of NO = 200 ppmv and a space velocity of 5000 h^−1^, the average specific capacity increases from 3.8 to 169.1 mg/g when the temperature decreases from 80 to – 20 ℃. For NO–O_2_ co-adsorption, the specific capacity increases along the adsorption bed due to the increasing NO_2_ concentrations. The adsorption capacity of SO_2_ is also significantly enhanced at cold temperatures. With an initial concentration of SO_2_ = 1000 ppmv, the specific capacity increases from 12.9 to 123.1 mg/g when the temperature decreases from 80 to – 20 ℃. A novel low-temperature adsorption (LAS) process is developed to simultaneously remove SO_2_ and NO_*x*_ from flue gas with a target of near-zero emission. A pilot-scale testing platform with a flue gas flowrate of 3600 Nm^3^/h is developed and tested. Emission of both SO_2_ and NO_*x*_ is less than 1 ppmv, and the predicted energy penalty is about 3% of the net generation.

## Introduction

SO_2_ and NO_*x*_ in flue gas are major air pollutants responsible for acid rain and photochemical smog. SO_2_ is an acidic gas and can be scrubbed by alkalic solutions such as lime, sodium carbonate, ammonia, etc^1,2^. Seawater is also considered as a scrubbing agent for desulfurization^3,4^. NO_*x*_ is composed of various forms of nitrogen oxides such as NO, NO_2_, N_2_O, etc. The dominant species is nitrogen monoxide (NO) which can be either reduced to N_2_ by selective catalytic reduction or oxidized to NO_2_ which can be scrubbed by alkalic solutions^5^. Wet flue gas desulfurization (WFGD) and SCR dinitrification are the dominant technologies in power plants nowadays.

**Figure 1:**
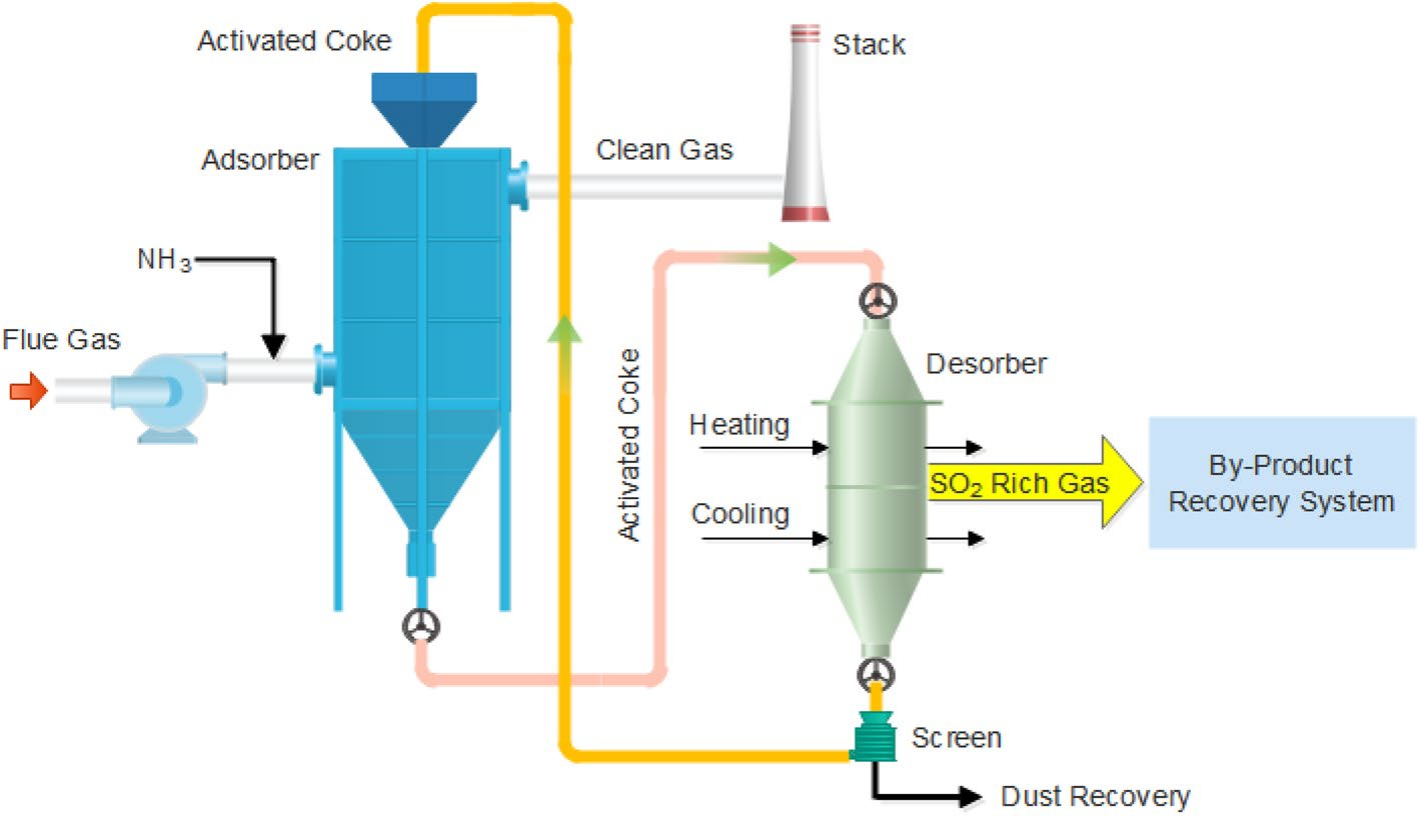
Schematic drawing of the activated coke desulfurization and denitrification process.

In addition, adsorption technology has also been widely used for gas cleanup. Simultaneous removal of SO_2_ and NO_*x*_ by activated carbon or coke has been successfully demonstrated in flue gas treatment^6,7^. An schematic drawing of the process is shown in Fig. 1.

Activated coke technology can remove SO_2_, NO_*x*_, Hg and other adsorbable pollutants simultaneously^8^. The removal of SO_2_ over activated carbon in the presence of oxygen and moisture involves a series of reactions that leads to the formation of sulfuric acid. The used carbon is regenerated through heating to recover their adsorbing activity. The desorbed SO_2_ is recycled as elemental sulfur, sulfuric acid or liquid SO_2_, as shown in Fig. 1. And the desorbed Hg and other trace contaminant species can be separated and collected during the sulfuric acid or sulfur production process. The typical operating temperature is 80–150 ℃ in the adsorber and 350–450 ℃ in the regenerator. The overall adsorption and desorption reactions are as follows^9^:

1 $$ 2{\text{SO}}_{2} + {\text{O}}_{2} + 2{\text{H}}_{2} {\text{O}} + 2{\text{C}}* \to 2{\text{C}}*{\text{H}}_{2} {\text{SO}}_{4} $$

2 $$ 2{\text{H}}_{2} {\text{SO}}_{4} + {\text{C}} \to 2{\text{SO}}_{2} {\text{ + CO}}_{2} + 2{\text{H}}_{2} {\text{O}} $$

For activated coke technology, NO_*x*_ is not removed by adsorption. It is removed through catalytic reduction over activated carbon by reacting with injecting NH_3_, which converts NO_*x*_ to non-toxic gas N_2_. The overall reactions are as follows^10^:

3 $$ 4{\text{NO}}{ + }4{\text{NH}}_{3} { + }{\text{O}}_{2} \to 4{\text{N}}_{2} { + }6{\text{H}}_{2} {\text{O}} $$

4 $$ 6{\text{NO}}_{2} + 8{\text{NH}}_{3} \to 7{\text{N}}_{2} + 12{\text{H}}_{2} {\text{O}} $$

Activated coke technology has been widely used for sintering flue gas treatment in iron and steel industry^11^, but only a few applications in power plants have been reported^12^. Power plant flue gas normally has a much higher concentration of SO_2_ and larger flowrate than sintering flue gas, the specific capacity of SO_2_ is not sufficient enough to treat the power plant flue gas. To improve the adsorption capacity, researchers have also investigated the possibility of using other adsorbents such as modified activated carbon^13,14^, activated carbon fibers^15^, molecular sieve^16,17^, alumina substrate impregnated with sodium carbonate^18^, copper oxide^19^, etc. But none of these adsorbents has been successfully commercialized.

Another defective feature of traditional activated coke technology is that NO_*x*_ is not able to be removed effectively through adsorption and the injection of ammonia is required to improve the denitrification rate. The removal rare of NO_*x*_ is less than 20% without NH_3_ and can be increased to around 70% if sufficient NH_3_ is injected^7,20^.

This study proposed a novel low-temperature adsorption (LAS) technology which is able to remove both SO_2_ and NO_*x*_ through adsorption with extraordinary adsorption capacity and high efficiency. The development of LAS technology is inspired by an interesting phenomenon observed accidentally that NO_*x*_ can be adsorbed by activated carbon effectively with an astonishing capacity when flue gas is cooled to cold temperatures. The fundamental behaviors of NO and SO_2_ adsorption at cold temperatures over activated carbon is investigated in this study. Furthermore, a pilot-scale testing platform with a flue gas treatment capacity of 3600 Nm^3^/h is designed and built to validate the performance of LAS technology. A brief introduction and first-hand data from the pilot-scale testing facility is also shared in this study. But the detail results from the pilot tests will be discussed in the future.

A comparison between the LAS technology and traditional activated coke technology is given in Table 1. The specific capacity is obtained in this study. The removal rate of traditional activated coke technology is from reported literature^7^ and the removal rate of LAS technology is obtained from the pilot platform.

**Table 1:** Comparison of LAS technology and traditional activated coke technology.

Items	Traditional activated coke technology	Low-temperature adsorption technology
Adsorption temperature	80–150 ℃	− 20–5 ℃
SO_2_ removal method	Adsorption	Adsorption
NO_*x*_ removal method	Catalytic reduction	Adsorption
Specific capacity of SO_2_	11.31 mg/g at 80 ℃	147.61 mg/g at − 20 ℃
Specific capacity of NO_*x*_	0.27 mg/g at 80 ℃	13.24 mg/g at – 20 ℃
SO_2_ removal rate	90–98%	≧ 99.9%
NO_*x*_ remove rate	70–80% (with NH_3_ injection)	≧ 99%
Injection of NH3	Required	Not required
Flue gas cooling system	Not required	Required
Adsorber	Large	Small

## Method

### Material

**Figure 2:**
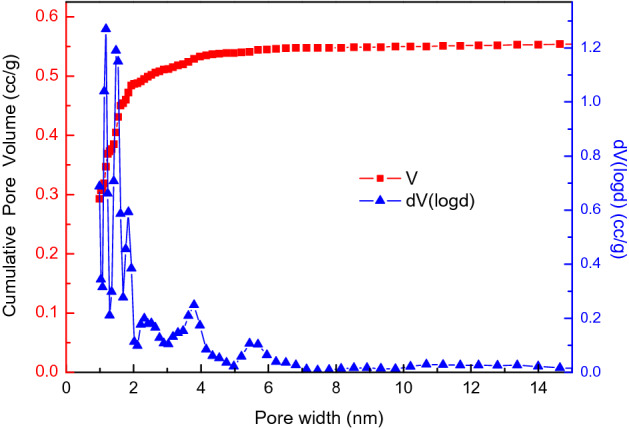
Pore distribution of coconut activated carbon (V: pore volume; dV: reciprocal of volume).

Commercial coconut activated carbon (CAC) with granular size between 26 and 30 mesh is used in this study. The surface physical properties of CAC is characterized by BET method (Quantachrome QUDRASORB SI). The specific surface area of CAC is 1314.5 m^2^/g, the pore size is mainly smaller than 2 nm, as shown in Fig. 2, in which DFT method is used to determine the pore size distribution. CAC is pretreated by heating to 300 ℃ in an vacuum tube for 2 h before each test. Mass of CAC is measured after the pretreatment. The loading density of granular CAC is 0.5 g/cm^3^.

### Experimental setup

**Figure 3:**
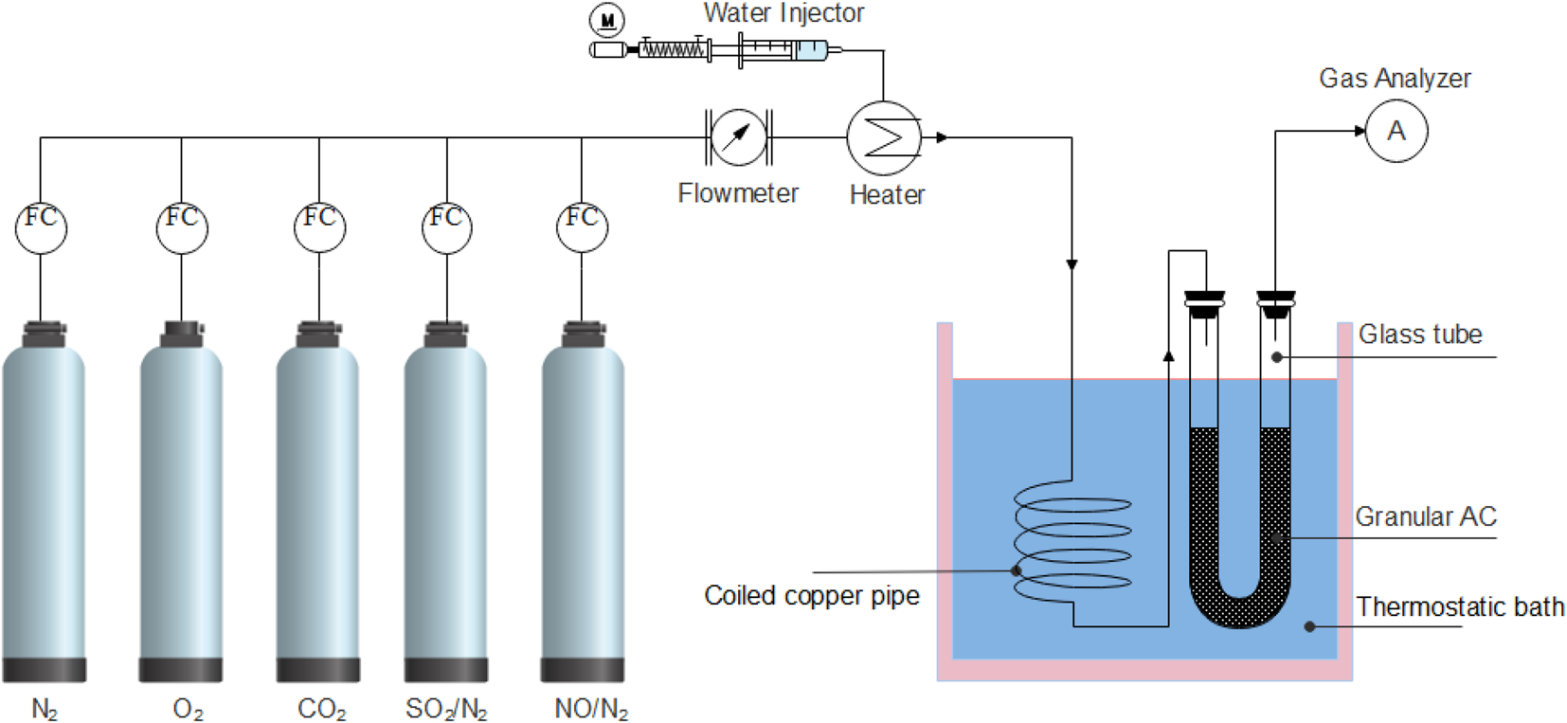
Experimental setup for low-temperature adsorption of SO_2_ and NO.

Figure 3 is a schematic drawing of the experimental setup for investigating the adsorption behaviors of SO_2_ and NO at cold temperatures. The dry flue gas has a volume flow rate of Q = 1 L/min, and has a volume concentration of N_2_ = 82%, O_2_ = 6%, CO_2_ = 12%, SO_2_ = 1000 ppmv and NO = 200 ppmv. Moisture in flue gas is added by a water injector controlled by a stepper motor. At 80 ℃, 10 vol.% of H_2_O is added to the flue gas. At 20, 0 and – 20 ℃, the concentration of H_2_O is reduced to the saturated moisture content at corresponding temperatures, which are 2.3, 0.6 and 0.1 vol.%, respectively. The is because the extra moisture will be removed from the flue gas during cooling process. The flue gas in pre-heated or pre-cooled to adsorption temperature by a coiled copper pipe immersing in a thermostatic bath with a temperature range of – 40 to 100 ℃. The granular activated carbon is loaded in a glass tube with an inner diameter of 5 mm which is also immersed in the thermostatic both. The difference between bath temperature and the gas temperature at the exit of adsorption bed is less than 2 ℃. During each test, 6 g of CAC is loaded and the space velocity of adsorption bed is 5000 h^−1^. The gas composition of flue gas leaving the adsorption tube is measured by flue gas analyzer testo 350. The gas analyzer is capable of measuring O_2_, CO_2_, SO_2_, NO and NO_2_. The method, accuracy and resolution of the analyzer for each component is given in Table 2.

**Table 2:** Basis information of gas analyzer TESTO 350.

Component	Method	Range	Accuracy	Resolution
CO_2_	IR sensor	0–25 vol.%	± 0.3 vol.%	0.01 vol.%
O_2_	Electrochemical sensor	0–25 vol.%	± 0.2 vol.%	0.01 vol.%
SO_2_	Electrochemical sensor	0 ~ +5000	± 5% of measured value	1 ppmv
NO	Electrochemical sensor	0 ~ +4000 ppmv	± 5% of measured value	1 ppmv
NO_2_	Electrochemical sensor	0 ~ +500 ppmv	± 5% of measured value	0.1 ppmv

### Pilot platform

A pilot-scale test platform is designed to simultaneously remove SO_2_, NO_*x*_ and other adsorbable pollutants based on the novel low-temperature adsorption technology. The designed flue gas flowrate is 3600 Nm^3^/h. The adsorption temperature is between − 20 to 5 ℃. The pollutants control target is near-zero emission: SO_2_ and NO_*x*_  ≦ 1 ppmv.

**Figure 4:**
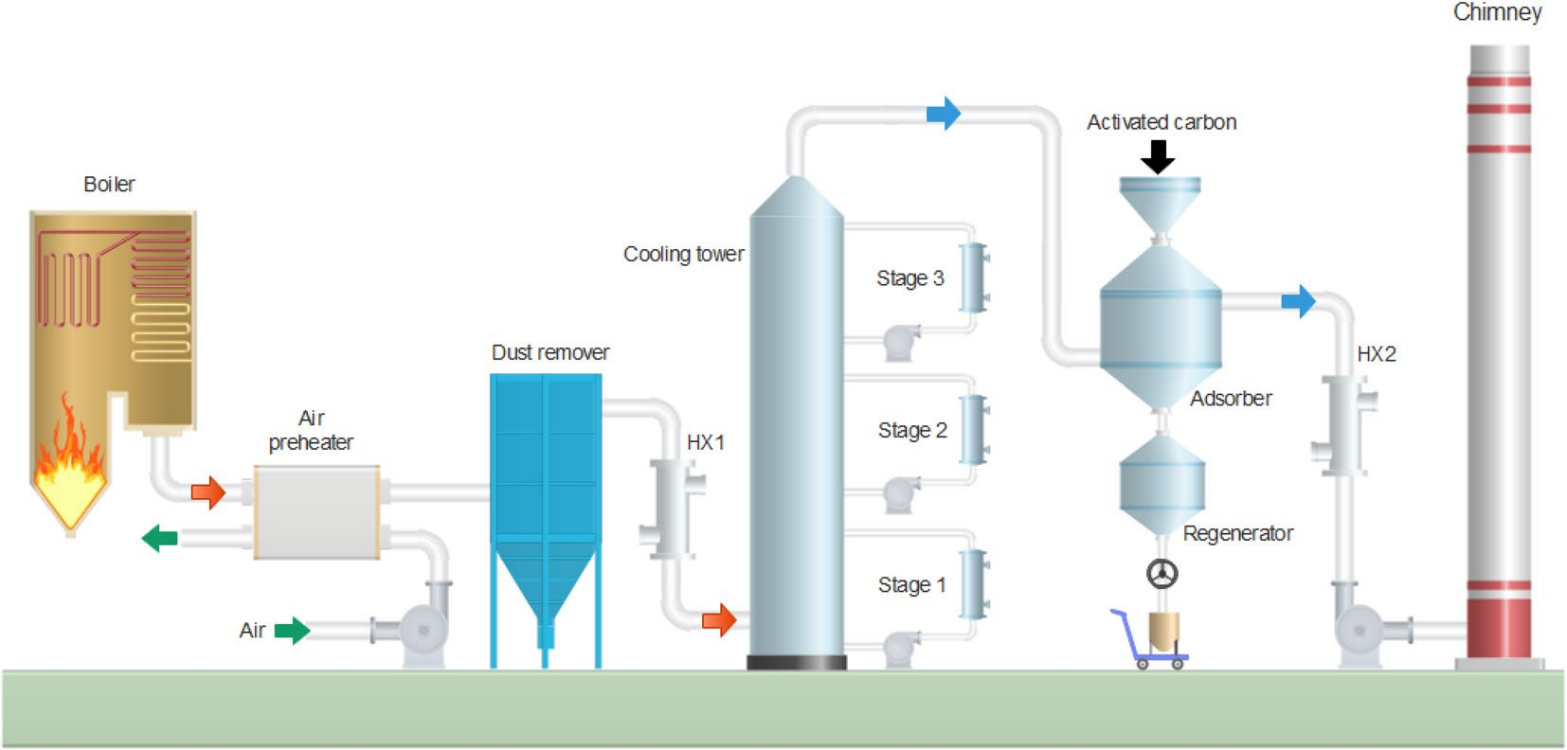
Schematic flowchart of pilot-scale test platform.

A schematic flowchart of the pilot-scale test platform is shown in Fig. 4. Flue gas is extracted from the inlet duct before SCR denitrification system. Hot flue gas is cooled to around 120 ℃ by an air preheater and the dust is then removed by a bag-type dust remover. Flue gas is further cooled to around 70 ℃ by a residue heat recovery exchanger (HX1) which can generate usable hot water. Flue gas is cooled to cold temperatures by a direct contact cooling (DCC) tower which has three cooling stages. In the lower stage (stage 1), flue gas is cooled close to room temperature by water scrubbing and the cooling load is provided by cooling water; in the second stage (stage 2), flue gas is cooled to 2–5 ℃ by cold water scrubbing and the cooling load is provided by an industrial chiller; in the upper stage (stage 3), flue gas is cooled to below freezing point by calcium chloride solution scrubbing and the cooling load is provided by an industrial refrigerator. The cold flue gas enters the adsorber in which SO_2_ and NO_*x*_ are removed by activated carbon through adsorption. The cold energy of clean flue gas is recovered by cooling the scrubbing water in the DCC cooling tower through HX2. The saturated activated carbon is heated in the regenerator to recover its adsorption activity for repeatable utilization. The desorbed SO_2_ can be recovered as elemental sulfur or sulfuric acid, and the desorbed NO_*x*_ can be introduced to the boiler to form a stable thermal balance of NO_*x*_–N_2_–O_2_, which has been successfully demonstrated in the NO_*x*_ SO process^18^. In this pilot study however, the post-treatment of desorbed gas is not considered.

## Results and discussion

### Phenomenon

This section is to introduce an interesting observation on the adsorption of SO_2_ and NO at cold temperatures, which to our knowledge has not yet been reported in open literature. This phenomenon is the origin of this study as well as the development of LAS technology.

The adsorption characteristics of SO_2_ and NO at 80, 20 and − 20 ℃ is investigated by the experimental setup shown in Fig. 3. The simulated flue gas has a composition described in section “Experimental setup”. During each test, 6 g of CAC is loaded in the tube and the space velocity is 5000 h^−1^. The concentration of SO_2_ and NO is 1000 and 200 ppmv, respectively. The dry flue gas has a volume flow rate of 1 L/min. The water content is 10 vol.% at 80 ℃, 2.3 vol.% (saturated) at 20 ℃, 0.1 vol.% (saturated) at – 20 ℃, respectively.

**Figure 5:**
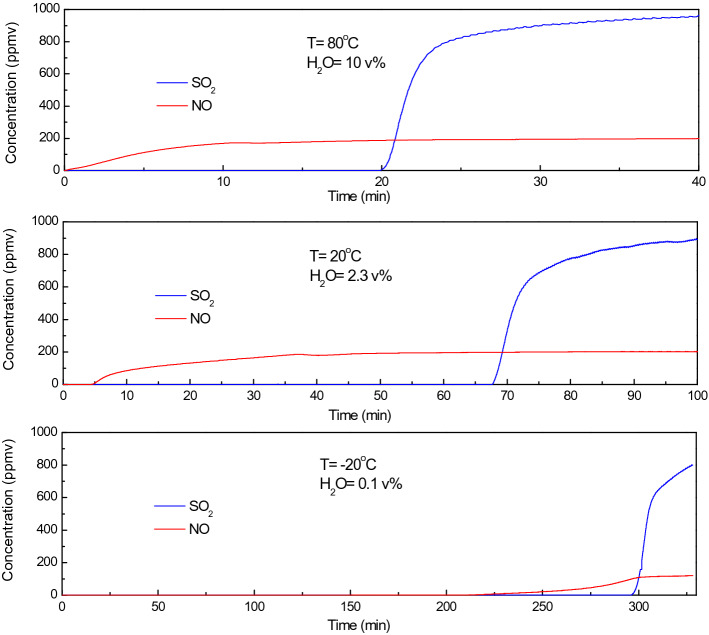
Breakthrough curves of SO_2_ (blue) and NO (red) adsorption at 80, 20 and − 20℃ (Simulated flue gas: SO_2_ = 1000 ppmv, NO = 200 ppmv, O_2_ = 6 vol.%, CO_2_ = 12 vol.%, space velocity = 5000 h^−1^).

Figure 5 is the breakthrough curve of SO_2_ (blue) and NO (red) of the three experiments. The concentrations of SO_2_ and NO leaving the adsorption bed (*C*_*out*_) are measured and plotted. Since the gas analyzer has a resolution of 1 ppmv for both SO_2_ and NO, the breakthrough time (*t*_*b*_, min) is defined as the time when *C*_*out*_(SO_2_) = 1 ppmv and *C*_*out*_(NO) = 1 ppmv. The breakthrough adsorption capacity (*A*_*b*_, mg/g) is defined as the adsorption capacity which can maintain near-zero emission (less than 1 ppmv). The saturated adsorption capacity (*A*_*s*_, mg/g) is defined as the maximum adsorption capacity when the bed stops adsorption. These two specific capacities are key parameters which determine the loading space velocity of adsorption bed and the frequency of bed regeneration.

The breakthrough and saturated adsorption capacity are calculated by the following equations:

5 $$ A_{b} = \frac{{C_{in} \times t_{b} \times Q \times M}}{22.4 \times m} \times 10^{ - 3} $$

6 $$ A_{s} - \frac{{\int_{{t_{0} }}^{{t_{e} }} {\left( {C_{in} - C_{out} (t)} \right)} \times Q \times M \times dt}}{22.4 \times m} \times 10^{ - 3} $$

where *A*_*b*_ and *A*_*s*_ are the breakthrough and saturated adsorption capacity (mg/g), respectively. *t*_*b*_(min) is the breakthrough time. *Q* (L/min) is the flow rate of simulated flue gas, which is 1 L/min. *M*(g/mol) is the molecular weight of SO_2_ or NO. *m*(g) is the mass of loaded CAC, which is 6 g when the space velocity is 5000 h^−1^. *C*_*in*_ (ppmv) is the inlet concentration, which is 1000 ppmv for SO_2_ and 200 ppmv for NO. *C*_*out*_ (t) is the outlet concentration of SO_2_ or NO at time *t*(min). In the time integral of Eq. (6), *t*_0_ is the starting time and *t*_*e*_ is ending time when the bed stops adsorption (*C*_*out*_ = *C*_*in*_).

As shown in Fig. 5, NO breaks through the adsorption bed almost instantaneously at an adsorption temperature of 80 ℃ which is close to the operating temperature of traditional activated coke technology. The breakthrough time of NO is about 5 min at 20 ℃ and 225 min at – 20 ℃. The breakthrough time of SO_2_ is 20, 68 and 295 min at 80, 20 and – 20 ℃, respectively. The specific capacity of SO_2_ and NO calculated by Eqs. (5) and (6) is given in Table 3.

**Table 3:** Adsorption capacity of SO_2_ and NO co-adsorption over CAC at various temperatures (simulated flue gas: SO_2_ = 1000 ppmv, NO = 200 ppmv, O_2_ = 6 vol.%, CO_2_ = 12 vol.%, space velocity = 5000 h^−1^).

Temperature (℃)	NO			SO_2_		
	*t*_*b*_ (min)	*A*_*b*_ (mg/g)	*A*_*s*_ (mg/g)	*t*_*b*_ (min)	*A*_*b*_ (mg/g)	*A*_*s*_ (mg/g)
80	0.05	0.002	0.27	20	9.52	11.31
20	5	0.22	0.80	68	32.38	37.18
− 20	225	10.07	13.24	295	140.47	147.61

Based on the observation from Fig. 5 and Table 3, it is found that the specific capacity of both NO and SO_2_ increases substantially with decreasing temperature. At above room temperature, the specific capacity of NO is less than 1 mg/g, which is insufficient for adsorption removal. At – 20 ℃, the specific capacity of NO increases to 13.24 mg/g, and the removal of NO_*x*_ from flue gas through adsorption becomes possible. At above room temperatures, the breakthrough time of NO is much shorter than that of SO_2_. At – 20 ℃, although the specific capacity of NO is far smaller than that of SO_2_, the breakthrough time of NO is close to that of SO_2_. This is because the concentration of SO_2_ is much higher than NO in the flue gas. Therefore, it is technically feasible to remove SO_2_ and NO_*x*_ by adsorption at cold temperatures.

The above experiments reveal the phenomenon of simultaneous adsorption of NO and SO_2_ in simulated flue gas. To understand the mechanism of NO and SO_2_ adsorption at cold temperatures, the adsorption of NO and SO_2_ are studied and discussed separately in sections “Adsorption of NO” and “Adsorption of SO_2_”.

### Adsorption of NO

**Figure 6:**
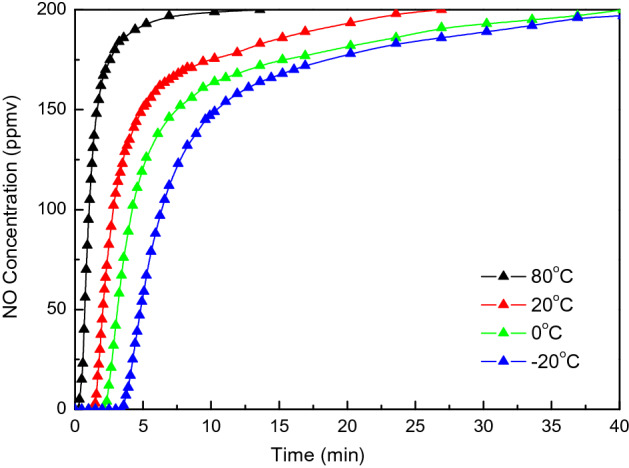
Breakthrough curve of NO adsorption at 80, 20, 0 and − 20℃ (NO = 200 ppmv, space velocity = 5000 h^−1^).

Figure 6 shows the breakthrough curves of the NO adsorption over CAC at 80, 20, 0 and − 20 ℃. 6 g of pretreated CAC is loaded in the adsorption tube and the space velocity is 5000 h^−1^. The inlet gas has flow rate of 1 L/min, and is composed of NO and N_2_, with an concentration of *C*_*in*_ (NO) = 200 ppmv. The concentration of NO at the outlet of adsorption bed is measured and plotted in Fig. 6. The breakthrough time and adsorption capacity of NO adsorption is given in Table 4. The physisorption (Van der Waals adsorption) of NO over activated carbon is enhanced by decreasing the adsorption temperature since physisorption is an exothermic process^21^. Without the presence of oxygen, the specific capacity of NO physisorption is less than 0.5 mg/g even at – 20 ℃.

**Table 4:** Adsorption characteristics of NO and NO–O_2_ over CAC at various temperatures (NO = 200 ppmv, O_2_ = 6 vol.%, space velocity = 5000 h^−1^).

Temperature (℃)	NO adsorption			NO–O_2_ co-adsorption			
	*t*_*b*_ (min)	*A*_*b*_ (mg/g)	*A*_*s*_ (mg/g)	*t*_*b*_ (min)	*A*_*b*_ (mg/g)	*A*_*s*_ (mg/g)	*η*(NO_2_) (%)
80	0.25	0.011	0.066	3.49	0.156	3.765	2.0
20	1.38	0.062	0.213	55.82	2.492	45.333	45.0
0	2.17	0.097	0.332	392.40	17.518	116.349	69.0
− 20	3.42	0.153	0.434	1591.75	71.061	169.142	91.5

**Figure 7:**
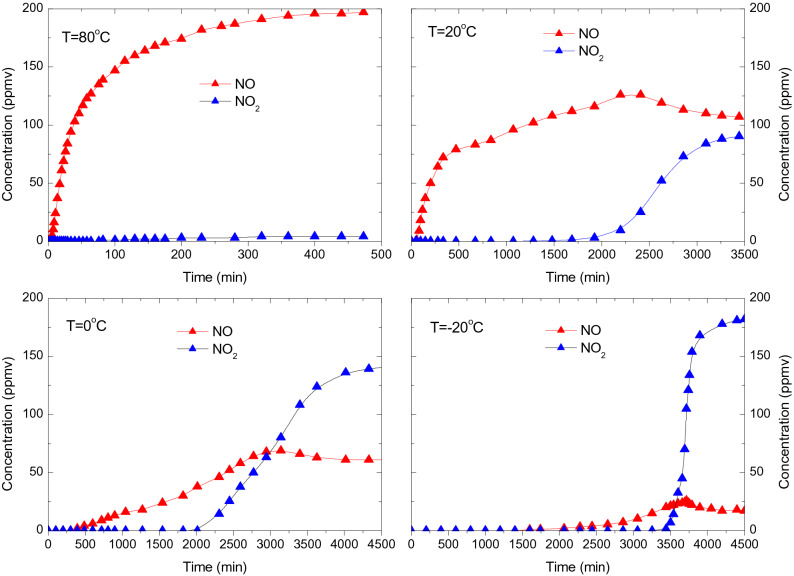
Breakthrough curves of NO (red) and NO_2_ (blue) during NO–O_2_ co-adsorption at 80, 20, 0 and – 20 ℃ (NO = 200 ppmv, O_2_ = 6 vol.%, space velocity = 5000 h^−1^).

Figure 7 shows the breakthrough curves of the NO-O_2_ co-adsorption over CAC at 80, 20, 0 and − 20 ℃. 6 g of pretreated CAC is loaded in the adsorption tube and the space velocity is 5000 h^−1^. The inlet gas has flow rate of 1 L/min, and is composed of NO (200 ppmv), O_2_ (6 vol.%) and N_2_. With the presence of oxygen, NO can be oxidized to NO_2_ over activated carbon^22,23^, a steady NO–NO_2_ equilibrium will be formed at the exit of activated carbon bed^24,25^. Therefore, the concentration of both NO and NO_2_ at the outlet of adsorption bed is measured and plotted in Fig. 7. At 80 ℃, NO (red) breakthrough the adsorption bed within a few minutes and reaches a steady concentration after about 400 min, and about 2% of NO is oxidized to NO_2_. At 20 ℃, NO (red) breakthrough the bed after about 56 min, and NO_2_ (blue) is detected after about 1600 min. After about 3500 min, both NO and NO_2_ reaches a steady concentration and the oxidation rate of NO is about 45%. When adsorption temperature is lowered to 0 ℃ and − 20℃, the breakthrough time of NO increases to 392 and 1591 min, respectively. Meanwhile, the oxidation rate of NO increases to 69% and 91.5%, respectively. For each test, the sum of NO and NO_2_ concentration is 200 ppmv when the steady state is reached.

The adsorption capacity of NO–O_2_ co-adsorption at various temperatures is given in Table 4. Both NO and NO_2_ has a breakthrough time during NO–O_2_ co-adsorption, as shown in Fig. 7. The breakthrough time for NO–O_2_ co-adsorption is defined as the breakthrough of NO which always occurs first. The breakthrough adsorption capacity *A*_*b*_ is calculated by Eq. (5) and the saturated adsorption capacity *A*_*s*_ is calculated by Eq. (6), where *C*_*out*_ (t) = *C*_*out*_ (NO_*x*_) = *C*_*out*_ (NO) + *C*_*out*_ (NO_2_) in the case of NO–O_2_ co-adsorption.

As shown in Table 4, the presence of oxygen can significantly increase the breakthrough time and specific capacity of NO. With presence of oxygen, NO can be oxidized to NO_2_ which is a much more adsorbable species over activated carbon^26,27^. The oxidation rate *η*(NO_2_) is calculated by the following equation:

7 $$ \eta \left( {{\text{NO}}_{2} } \right) = \frac{{C_{out} \left( {{\text{NO}}_{2} } \right)}}{{C_{in} \left( {{\text{NO}}} \right)}} \times 100\% $$

where *C*_*out*_ (NO_2_) is the concentration of NO_2_ when it reaches steady state. *C*_*in*_ (NO) is the inlet NO concentration. The oxidation rate of NO at 80, 20, 0 and – 20 ℃ is given in Table 4 as well. With the presence of oxygen, the catalytic oxidation of NO is significantly enhanced by at sub-zero temperatures. The mechanism of NO oxidation over activated carbon is complicated, involving both surface reactions and gaseous reactions. The oxidation of gaseous NO by adsorbed oxygen over the activate surface site is believed to be the dominant pathway^26^.

8 $$ 2{\text{C}}\left( \, \right) + {\text{O}}_{2} + \to \, 2{\text{C}}\left( {\text{O}} \right) $$

9 $$ {\text{C}}\left( {\text{O}} \right) + {\text{NO}} \to {\text{C}} - {\text{NO}}_{2} \,\,{\text{or}}\,{\text{C}} - {\text{ONO}} \to {\text{C}} + {\text{NO}}_{2} $$

where C( ) represents the activated carbon with active surface site. Based on reaction (9), the oxidation reaction rate can be calculated by the following equation:

10 $$ \frac{{d\left[ {{\text{NO}}_{2} } \right]}}{dt} = k\left( T \right)\left[ {{\text{C}}\left( {\text{O}} \right)} \right] \times \left[ {{\text{NO}}} \right] $$

Temperature can impact the oxidation reactions in many ways. First of all, the physisorption of oxygen over activated carbon is enhanced and the concentration of C(O) is increased by reducing adsorbing temperature^28^. Secondly, the rate constant *k*(*T*) of NO oxidation increases with decreasing temperature^29^.

As shown in Table 4, temperature has a significant impact on the breakthrough time and specific capacity of NO–O_2_ co-adsorption. When the adsorption temperature decreases from 80 to – 20 ℃, the breakthrough time increases from 3.5 to 1591 min, and the saturated capacity increases from 3.8 to 169 mg/g. This extraordinary increase of specific capacity is due to the NO_2_ adsorption.

**Figure 8:**
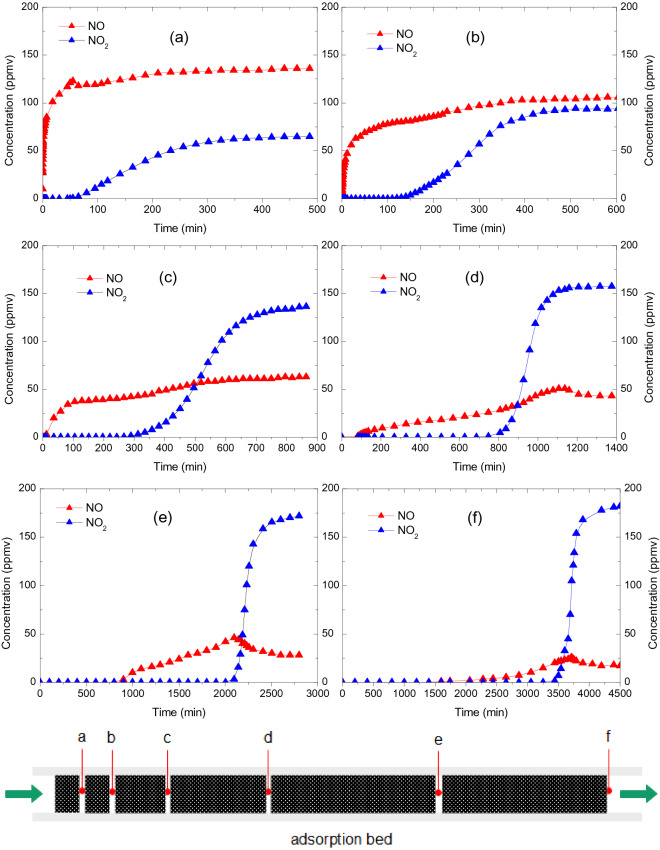
Breakthrough curves of NO (red) and NO_2_ (blue) during NO–O_2_ co-adsorption at various cross sections of adsorption bed: (**a**) 120,000 h^−1^, (**b**) 60,000 h^−1^, (**c**) 30,000 h^−1^, (**d**) 15,000 h^−1^, (**e**) 7500 h^−1^ and (**f**) 5,000 h^−1^ (NO = 200 ppmv, O_2_ = 6 vol.%, T = − 0℃).

Figure 8 shows the breakthrough curves of the NO–O_2_ co-adsorption over CAC with various loading space velocity. In Fig. 8a, 0.25 g CAC is loaded and the space velocity is 120,000 h^−1^; in Fig. 8b, 0.5 g CAC is loaded and the space velocity is 60,000 h^−1^; in Fig. 8c, 1 g CAC is loaded and the space velocity is 30,000 h^−1^; in Fig. 8d, 2 g CAC is loaded and the space velocity is 15,000 h^−1^; in Fig. 8e, 4 g CAC is loaded and the space velocity is 7500 h^−1^; in Fig. 8f, 6 g CAC is loaded and the space velocity is 5000 h^−1^. All six experiments are conducted at – 20 ℃. The inlet gas has flow rate of 1 L/min, and is composed of NO (200 ppmv), O_2_ (6 vol.%) and N_2_.

The purpose of conducting these six independent experiments is to mimic the experiment of simultaneously motoring NO and NO_2_ at six different cross sections of a single adsorption bed which is difficult to achieve in our experimental setup.

**Figure 9:**
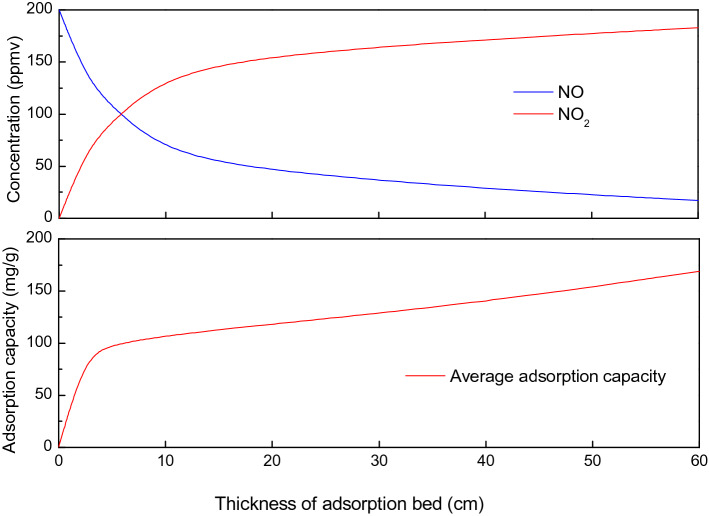
Oxidation and adsorption characteristics of NO–O_2_ along activated carbon bed (NO = 200 ppmv, O_2_ = 6 vol.%, T = − 20 ℃, space velocity = 5000 h^−1^ when thickness is 60 cm).

The average adsorption capacity and oxidation rate along the axial direction of the CAC bed are given in Fig. 9 and Table 5. As shown in Fig. 9, the NO_2_ concentration increases along the bed, indicating that the oxidation rate increases along the axial direction of the adsorption bed. At each cross section of the adsorption bed, a stable NO–NO_2_ equilibrium is formed both in the gas phase and the adsorption surface. Therefore the NO–O_2_ co-adsorption mechanism involves the adsorption of both NO and NO_2_ over activated carbon. Since NO_2_ is a much more adsorbable species than NO^30^, the adsorption capacity increases with the increasing NO_2_ concentration along the axial direction of activated carbon bed, as shown in Fig. 9. This is quite different with the adsorption of SO_2_ which has an uniform adsorption capacity along the adsorption bed. Due to this distinct characteristics, it should be noted that the adsorption capacity given in Fig. 9 and Table 5 should be defined as average adsorption capacity of a specific CAC bed. The real adsorption capacity at each cross section should be larger than the average value.

**Table 5:** Adsorption characteristics of NO-O_2_ at various CAC load (NO = 200 ppmv, O_2_ = 6 vol.%, T = − 20 ℃).

Mass of CAC (g)	Space velocity (h^−1^)	NO–O_2_ adsorption			
		*t*_*b*_ (min)	*A*_*b*_ (mg/g)	*A*_*s*_ (mg/g)	*η*(NO_2_) (%)
0.25	120,000	0.01	0.011	88.091	32.0
0.5	60,000	0.44	0.236	98.476	47.0
1	30,000	10.86	2.909	107.356	68.5
2	15,000	88.42	11.841	118.259	78.5
4	7,500	973.18	65.169	138.999	86.0
6	5,000	1591.75	71.061	169.142	91.5

Based on the above analysis, it is now quite clear why the breakthrough time and adsorption capacity of NO–O_2_ co-adsorption increases dramatically when the adsorption temperature is lowered to below room temperatures. At cold temperatures, the catalytic oxidation of NO is fastened, therefore the breakthrough time and breakthrough adsorption capacity are largely increased. In addition, the adsorption capacity of NO_2_ increases significantly at cold temperatures, leading to prominent increase of saturated adsorption capacity.

### Adsorption of SO_2_

**Figure 10:**
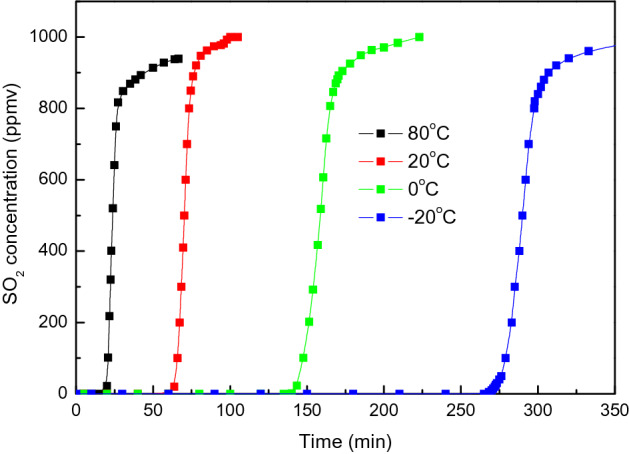
Breakthrough curve of SO_2_ adsorption over CAC at various temperatures (SO_2_ = 1000 ppmv, O_2_ = 6 vol.%, flow rate = 1 L/min, space velocity = 5000 h^−1^).

Figure 10 shows the breakthrough curves of the SO_2_ adsorption over CAC at 80, 20, 0 and – 20 ℃. 6 g of pretreated CAC is loaded in the adsorption tube and the space velocity is 5000 h^−1^. The inlet gas has flow rate of 1 L/min, and is composed of SO_2_ (1000 ppmv), O_2_ (6 vol.%) and N_2_. The concentration of SO_2_ at the outlet of adsorption bed is measured and plotted in Fig. 10. Results indicate that the breakthrough time and specific capacity of SO_2_ adsorption increases with decreasing temperature. When the temperature decreases from 80 to – 20 ℃, the breakthrough time and adsorption capacity increase by about 13 and 10 times, respectively. SO_2_ adsorption over activated carbon is fast and the breakthrough adsorption capacity is quite close to the saturated adsorption capacity. The breakthrough curve has a sharp slope which is quite different with that of NO adsorption.

The impact of O_2_, CO_2_ and H_2_O on the adsorption of SO_2_ at various temperatures are also investigated. As shown in Table 6, the presence of oxygen has a slight improvement of SO_2_ adsorption due to the catalytic oxidation over activated carbon. The presence of CO_2_ in the opposite has a negative impact on the SO_2_ adsorption due to the occupation of active carbon surface. The presence of H_2_O and O_2_ can enhance the adsorption of SO_2_ through H_2_SO_4_ adsorption^9^.

**Table 6:** Adsorption characteristics of SO_2_ over activated carbon (SO_2_ = 1000 ppmv, O_2_ = 6 vol.%, CO_2_ = 12 vol.%, space velocity = 5000 h^−1^) (H_2_O: 10 vol.%, 2.3 vol.% saturated, 0.6% saturated and 0.1 vol.% saturated at 80, 20, 0 and − 20 ℃).

Temperature (℃)	SO_2_		SO_2_–O_2_		SO_2_–O_2_–H_2_O		SO_2_–CO_2_	
	*t*_*b*_ (min)	*A*_*s*_ (mg/g)	*t*_*b*_ (min)	*A*_*s*_ (mg/g)	*t*_*b*_ (min)	*A*_*s*_ (mg/g)	*t*_*b*_ (min)	*A*_*s*_ (mg/g)
80	19	12.87	20	13.29	28	18.25	18	11.89
20	57	29.88	61	33.97	62	34.32	52	28.69
0	130	67.72	138	76.40	138	73.02	109	58.74
-20	235	123.11	265	140.32	269	140.17	188	108.27

**Figure 11:**
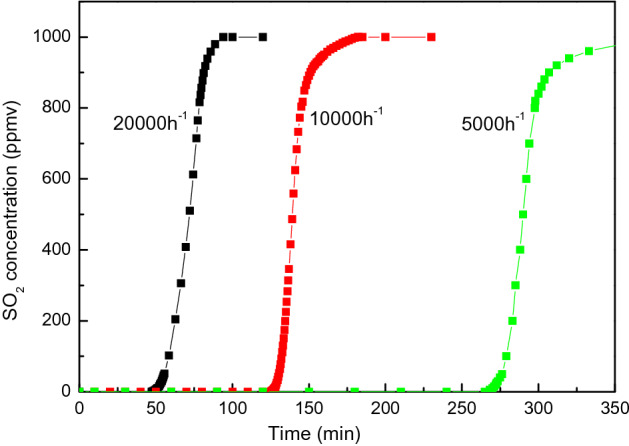
Breakthrough curve of SO_2_ adsorption over CAC at various space velocity (SO_2_ = 1000 ppmv, O_2_ = 6 vol.%, T = − 20 ℃, flow rate = 1 L/min).

The impact of space velocity of CAC load on the adsorption of SO_2_ is shown in Fig. 11. The breakthrough time is doubled when the CAC load is doubled. This indicates that the adsorption capacity (mg/g CAC) is irrelevant with space velocity and is a constant value at certain temperature and partial pressure of SO_2_. This is also quite different with the adsorption capacity of NO (with the presence of O_2_) which increases along the adsorption bed as shown in Fig. 9.

**Figure 12:**
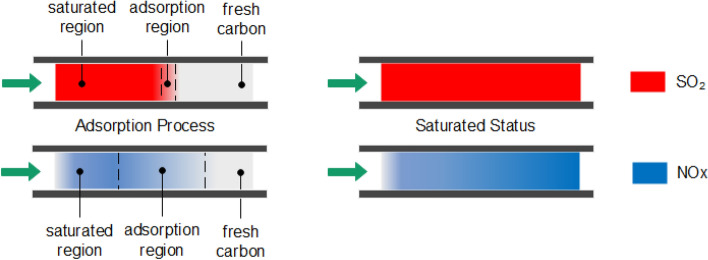
Adsorption process of SO_2_ (red) and NO (blue) with presence of oxygen over activated carbon.

The adsorption process of SO_2_ and NO with the presence of oxygen over activated carbon is illustrated in Fig. 12. There are three regions during the SO_2_ adsorption process (upleft). In the saturated region, an equilibrium of [SO_2_(g), SO_2_(a)] is established. Where (g) present gaseous phase and (a) represent adsorbed phase. In the adsorption region, SO_2_(g) is being adsorbed and converted to SO_2_(a). Since the adsorption of SO_2_ is fast, the adsorption region is within a narrow region. In the fresh carbon region, both SO_2_(g) and SO_2_(a) are zero. When the adsorption bed reaches the saturated status (upright), a homogeneous equilibrium of SO_2_ (g) and SO_2_ (a) is established cross the entire adsorption bed:

11 $$ {\text{SO}}_{2} \left( {\text{g}} \right) \rightleftharpoons {\text{SO}}_{2} \left( {\text{a}} \right) $$

For NO + O_2_ adsorption (lower left), due to the catalytic oxidation, equilibriums of [NO(g), NO(a)] and [NO_2_(g), NO_2_(a)] co-exist, with increasing NO_2_ and decreasing NO along the bed. Since the adsorption of NO is almost neglectable compared with NO_2_, the total adsorption capacity increases along the adsorption bed in the saturated region. In the adsorption region, the remaining NO is further oxidized to NO_2_ and adsorbed. Since the adsorption rate is limited by oxidation rate, the adsorption of NO + O_2_ is much slower than SO_2_ and the adsorption region is much wider. When it is reaches saturated status, equilibrium of [NO(g), NO(a)] with decreasing partial pressure and equilibrium of [NO_2_(g), NO_2_(a)] with increasing partial pressure are established along the adsorption bed. At each cross section of the bed, the following equilibriums co-exist:

12 $$ {\text{NO}}\left( {\text{g}} \right) \rightleftharpoons {\text{NO}}\left( {\text{a}} \right) $$

13 $$ {\text{NO}}_{2} \left( {\text{g}} \right) \rightleftharpoons {\text{NO}}_{2} \left( {\text{a}} \right) $$

14 $$ 2{\text{NO}}\left( {\text{g}} \right) + {\text{O}}_{2} \left( {\text{g}} \right) \rightleftharpoons 2{\text{NO}}_{2} \left( {\text{g}} \right) $$

15 $$ 2{\text{NO}}\left( {\text{a}} \right) + {\text{O}}_{2} \left( {\text{a}} \right) \rightleftharpoons 2{\text{NO}}_{2} \left( {\text{a}} \right) $$

### Pilot test

**Figure 13:**
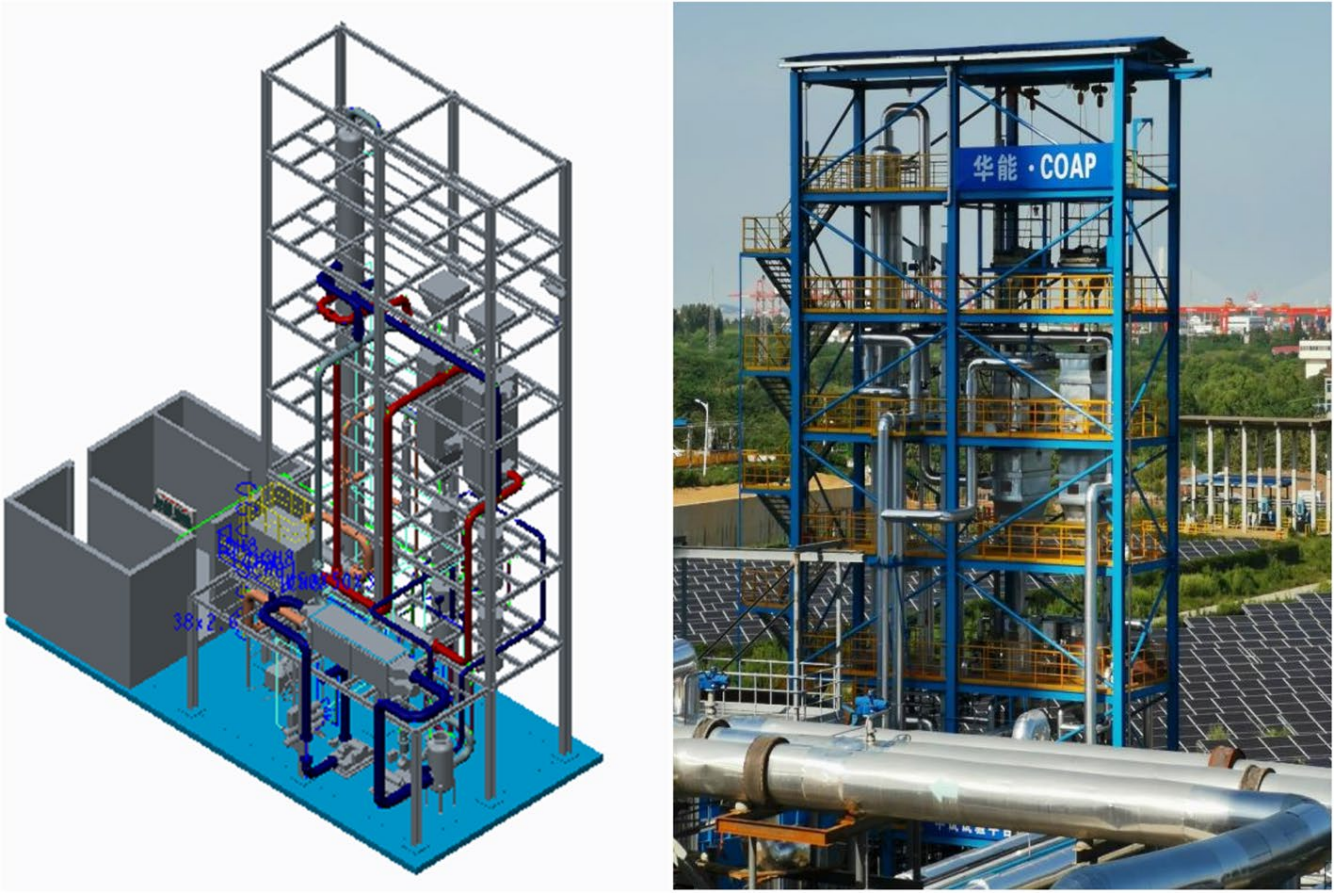
3D model (left) and a photo (right) of the pilot test platform.

**Figure 14:**
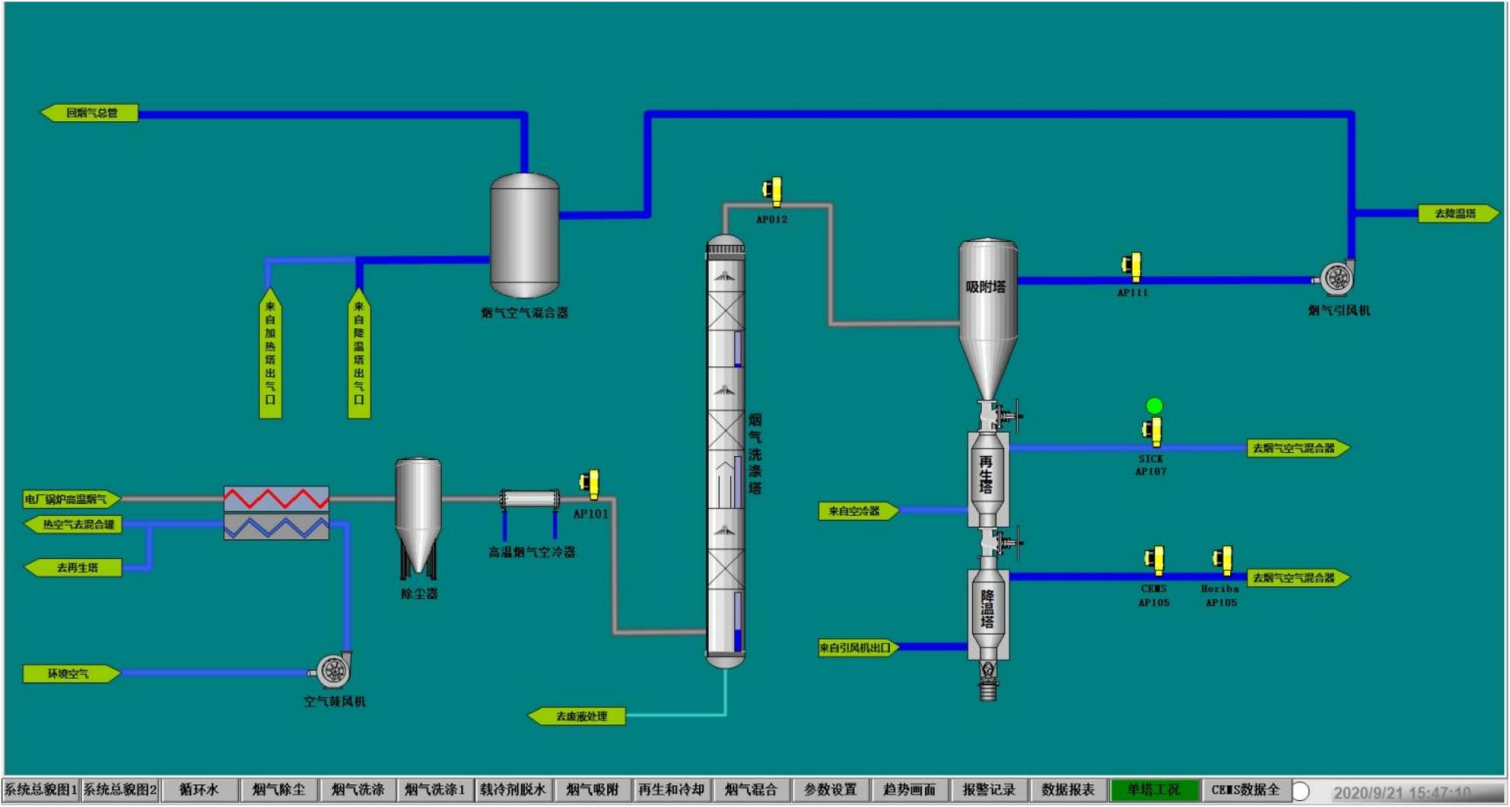
Process diagram of the low-temperature adsorption system.

**Figure 15:**
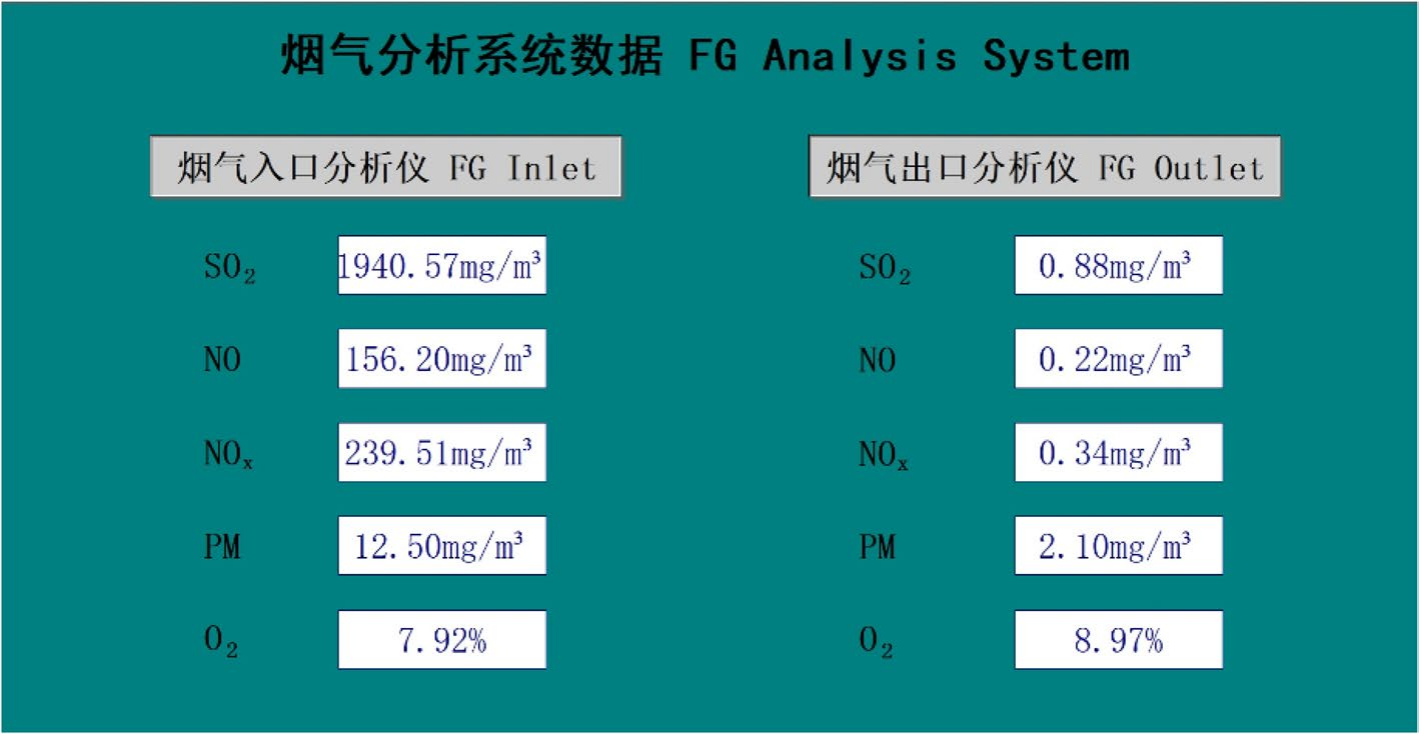
Inlet and outlet flue gas composition monitored by online CEMS system.

**Figure 16:**
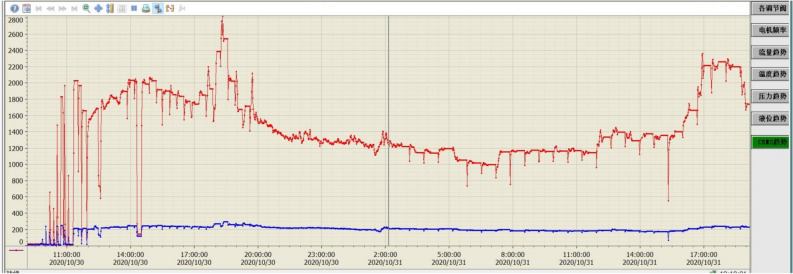
SO_2_ (red) and NO_*x*_ (blue) concentration of inlet flue gas during 72-h operation.

**Figure 17:**
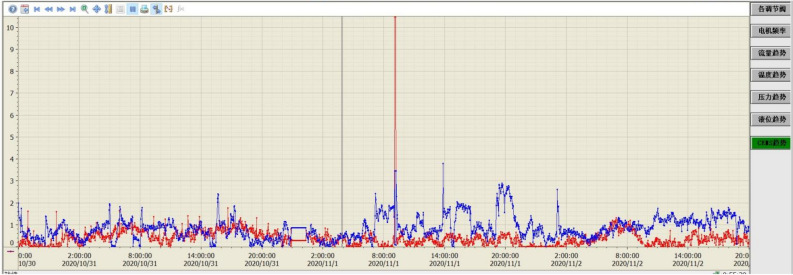
SO_2_ (red) and NO_*x*_ (blue) concentration of outlet flue gas during 72-h operation.

The pilot test platform is built in Huaneng Yueyang Power Plant and is accomplished in September 2020. A detail description of the process is given in section “Pilot platform”. A picture of the pilot test platform is shown in Fig. 13. A preliminary low-temperature adsorption test is conducted in October. The flue gas flow rate is 3600 Nm^3^/h and the operating temperature is − 15  to − 20 ℃. Figure 14 is simplified process diagram of the pilot system. Figure 15 shows the inlet and outlet flue gas composition monitored by online CEMS system. Figures 16 and 17 shows some of the operating data of a successive 72 h test. The inlet flue gas has an SO_2_ concentration of around 500–1000 ppmv and NO_*x*_ concentration of around 70–100 ppmv. The concentrations of both SO_2_ and NO_*x*_ are reduced to below 1 ppmv when leaving the adsorber. More tests are undergoing and will be shared as soon as the data are unclassified. In addition, the performance and energy penalty are evaluated by conducting Aspen Plus modeling, and the energy penalty is about 2–3% of the total net power generation depending on the ambient temperatures. The detail data of the pilot test and the modeling work will be presented and discussed shortly.

## Conclusion

In this study, the oxidation and adsorption characteristics of NO over activated carbon at cold temperatures is investigated. With the presence of oxygen, both the oxidation rate and adsorption of NO over activated carbon is enhanced significantly at cold temperatures. The breakthrough time of NO increases from 3.45 to 1591.75 min when the adsorption temperature decreases from 80 to −20 ℃. At each cross sections along the adsorption bed, NO and NO_2_ equilibrium is established with decreasing NO and increasing NO_2_ along the bed, which leads to a increasing adsorption capacity along the bed. The adsorption of SO_2_ also increases significantly at cold temperatures. The adsorption capacity increases from 12.87 to 123.11 mg/g when the temperature decreases from 80 to − 20 ℃. A novel low-temperature adsorption process is developed to simultaneously remove SO_2_ and NO_*x*_ from flue gas. A pilot scale test platform is built and the low-temperature adsorption process is tested. Near-zero emission of both SO_2_ and NO_*x*_ is achieved during a 72 h performance validation test.
